# The Extraction, Determination, and Bioactivity of Curcumenol: A Comprehensive Review

**DOI:** 10.3390/molecules29030656

**Published:** 2024-01-30

**Authors:** Jie Li, Yitian Sun, Guohua Li, Chunsong Cheng, Xinbing Sui, Qibiao Wu

**Affiliations:** 1 State Key Laboratory of Quality Research in Chinese Medicines, Faculty of Chinese Medicine, Macau University of Science and Technology, Macau 999078, China; 18256960482@sina.cn (J.L.);; 2 College of Pharmacy, Hangzhou Normal University, Hangzhou 311121, China; 3 School of Environmental and Chemical Engineering, Zhaoqing University, Zhaoqing 526061, China; 4 Lushan Botanical Garden, Chinese Academy of Sciences, Jiujiang 332900, China; 5 Zhuhai M.U.S.T. Science and Technology Research Institute, Zhuhai 519031, China; 6 Guangdong-Hong Kong-Macao Joint Laboratory for Contaminants Exposure and Health, Guangzhou 510006, China

**Keywords:** *Curcuma wenyujin*, curcumenol, biological activity, TCM

## Abstract

*Curcuma wenyujin* is a member of the *Curcuma zedoaria* (zedoary, Zingiberaceae) family, which has a long history in traditional Chinese medicine (TCM) due to its abundant biologically active constituents. Curcumenol, a component of *Curcuma wenyujin*, has several biological activities. At present, despite different pharmacological activities being reported, the clinical usage of curcumenol remains under investigation. To further determine the characteristics of curcumenol, the extraction, determination, and bioactivity of the compound are summarized in this review. Existing research has reported that curcumenol exerts different pharmacological effects in regard to a variety of diseases, including anti-inflammatory, anti-oxidant, anti-bactericidal, anti-diabetic, and anti-cancer activity, and also ameliorates osteoporosis. This review of curcumenol provides a theoretical basis for further research and clinical applications.

## 1. Introduction

**Figure 1:**
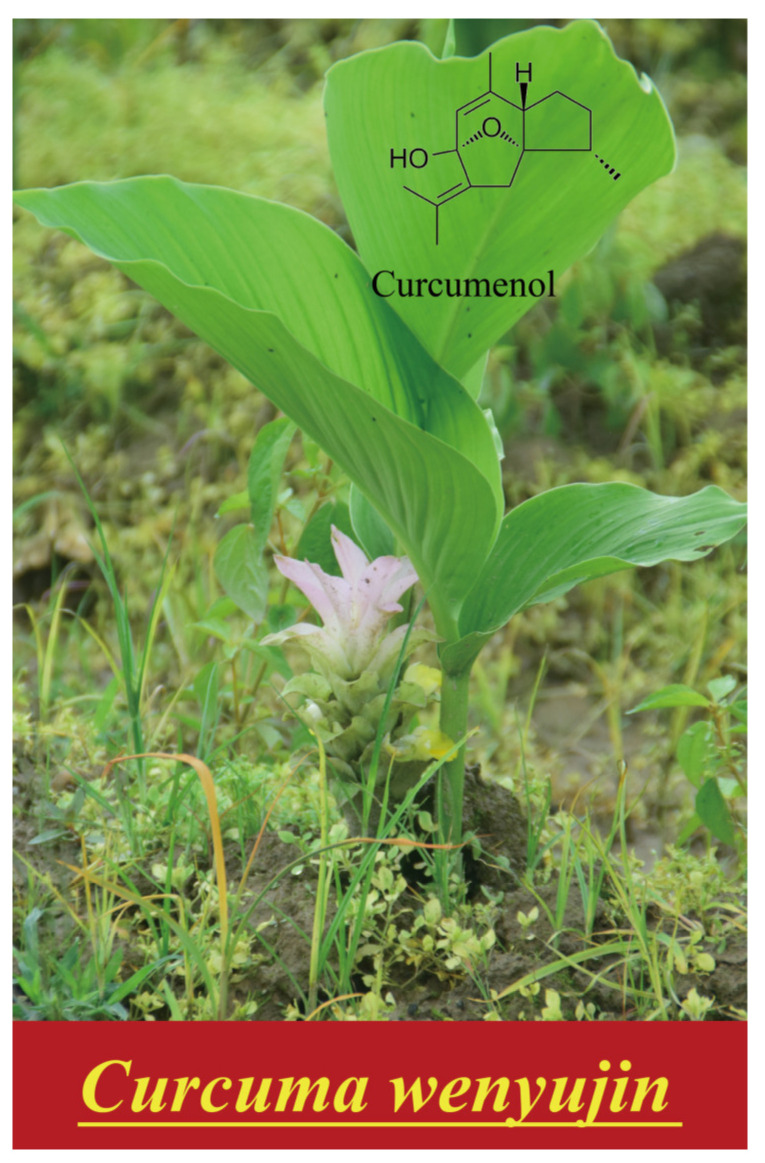
Picture of *Curcuma wenyujin* and the structure of curcumenol.

*Curcuma wenyujin* Y. H. Chen et C. Ling (*Curcuma wenyujin*) is a member of the *Curcuma zedoaria* (zedoary, Zingiberaceae) family, which is primarily distributed in Zhejiang Province, China [1]. It has a long history of extensive utilization in TCM. It is one of the members of the well-known TCM group named “Zhebawei”, which includes eight authentic Chinese herbal medicines from Zhejiang Province (*Atractylodes*, *paeony*, *Zhejiang fritillaria*, *Chrysanthemum morifolium*, *Corydalis glaucescens*, *Scrophularia ningpoensis Hemsl.*, *Ophiopogon japonicus (Thunb.) Ker-Gawl*, and *Wen Yujin*) [2] (Figure 1). *Curcuma wenyujin* can be incorporated into three different Chinese medicines, “Acruginous Turmeric Rhizome”, “Wenyujin”, and “Wenyujin Rhizoma Concisum”, and exhibits anti-inflammatory [3], anti-tumor [4], anti-oxidant [5], anti-bacterial, anti-viral, and hepatoprotective properties. The pharmacological value of *Curcuma wenyujin* is derived from its constituents, which have been reported to comprise a total of 169 compounds, thus far. These include monoterpenoids, sesquiternoids, diterpenoids, curcuminoids, etc. [6]. Curcumin, an active ingredient of *Curcuma wenyujin*, has been widely investigated and applied [7]. Curcumenol is one of the other sesquiterpenoid active ingredients (following the Chinese Pharmacopoeia), which has been extracted from the edible rhizome of *Curcuma zedoaria* since the 1960s [8]; it has been reported as one of the primary constituents in *Curcuma* plant essential oils, as well as an important component of food and traditional remedies [9,10,11,12]. A growing number of studies have suggested that it exhibits useful biological effects, including anti-cancer [13,14,15,16], anti-inflammatory [17,18,19], anti-bactericidal [20,21], and liver protection activities [22], among others. *Curcumina longa*, another important member of the *Curcuma zedoaria* (zedoary, Zingiberaceae) family, has also attracted research interest. The differences in the components and physiological effects of the two herbs are shown in Table 1.

Despite the huge pharmacological potential of curcumenol, its further application has been hindered by unsatisfactory physical properties (e.g., low water solubility). In this research, relevant studies and reports were reviewed to summarize the methods related to the extraction, determination, and pharmacology of curcumenol, with the aim of promoting the investigation and utilization of curcumenol in human healthcare.

**Table 1:** Differences between the components and physiological effects of *Curcuma wenyujin* and *Curcumina longa*.

*Curcuma wenyujin*		*Curcumina longa*
The Content of Unique Chemical Markers to Distinguish the Two Herbs [23]		
Curcumenol	>	Curcumenol
Curcumenone	>	Curcumenone
Neocurdione	>	Neocurdione
Curdione	>	Curdione
Curcumin	<	Curcumin
Physiological effects		
Anti-inflammatory [24]		Anti-inflammatory [25]
Anti-oxidant [26]		Anti-oxidant [27]
Antimicrobial [28] and antiviral activity [29]		Antimicrobial [30] and antiviral activity [31]
Anti-diabetic [32]		Anti-diabetic [33]
Hepatoprotective [34]		Hepatoprotective [35]
Effects on the cardiovascular system [36]		Effects on the cardiovascular system [37]
Analgesic effect [38]		Analgesic effect [39]
Effect on the nervous system: Alzheimer’s disease [40]		Effect on the nervous system: improve memory impairment [41]
Anti-cancer: glioma [42], breast cancer [43], liver cancer [44], gastric cancer [45], colon cancer [46], leukemia [47], cervical cancer [48], lung cancer [49]		Anti-cancer: liver cancer [50], colon cancer [51], cervical cancer [52], human thyroid cancer [53], human nasopharyngeal carcinoma cells [54], lung cancer [55]
		Effects on the respiratory system [56]
		Effect on the digestive system [57]
		Anticoagulant effect [58]

## 2. Traditional Medicinal Uses of Curcumenol-Related Herbs

*Curcuma* is an important herb in Chinese medicine, with a long history of use for about 1500 years. It has significance in healthcare due to its various medicinal effects. It has been reported that *Curcuma* can promote blood circulation, promote the flow of qi, unblock meridians, and relieve pain [1]. In-depth investigations have also been conducted to elucidate which exact compounds exert the abovementioned traditional curative effects, based on current medical theories. A series of compounds related to these treatment effects have been identified, including curcumenol. Curcumenol exhibits anti-inflammatory, anti-tumor, anti-virus, anti-oxidation, and hepatoprotective effects, as listed in Table 2. The above-described pharmacologica activity of curcumenol provides further evidence for the use of *Curcuma* in traditional medicines.

**Table 2:** Traditional uses of curcumenol-related herbal medicines.

Scientific Name	Traditional Name	Plant Part Used	Effective Compounds and Ref.	Traditional Medicine Use	Monotherapy or an Adjunct and Ref.
*Curcuma zedoaria*	Zedoary, Zingiberaceae	Rhizome	Curcumenol [17]	Osteoarthritis	An adjunct to allopathic medication [59]
*Curcuma zedoaria*	White turmeric	Rhizome	Curcumenol [18]	Anti-inflammatory	An adjunct to allopathic medication [60]
*Curcuma zedoaria*	White turmeric	Rhizome	Curcumenol and six other compounds [19]	Psoriasis	An adjunct to allopathic medication [61]
*Curcuma*	*Curcuma phaeocaulis*, *Curcuma kwangsiensis*, *Curcuma wenyujin*	Rhizome	Curcumenol, Curcumol, β-elemene, curdione [21]	Anti-fungal	An adjunct to allopathic medication [62]
*Curcuma zedoaria*	Zingiberaceae	Rhizome	Curcumenol and some principal sesquiterpenes [22]	Hepatoprotective	An adjunct to allopathic medication [63]
*Curcuma zedoaria*	Zingiberaceae	Rhizome	Curcumenol, dihydrocurdione [64,65]	Analgesic	An adjunct to allopathic medication [66]
*Curcuma*	Zingiberaceae	*Curcuma aromatica* Salisb. rhizome	Curzerene, isoprocurcumenol, and (+)-curcumenol [67]	Coronary heart disease	An adjunct to allopathic medication [68]
*Curcuma zedoaria*	Zingiberaceae	Rhizome	Curcumenol [69]	Osteoporosis	An adjunct to allopathic medication [70]
*Curcuma zedoary*		Rhizome	Curcumenol [71]	Liver cancer	An adjunct to allopathic medication [72]
*Curcuma*		Rhizome	Curcumenol [73]	Cervical cancer	An adjunct to allopathic medication [74]
*Curcuma wenyujin*	Wenyujin	Rhizome	Curcumenol [13]	Lung cancer	An adjunct to allopathic medication [75]
*Curcuma zedoaria rhizome*	Zingiberaceae	*C. zedoaria* rhizomes	Curcumenol, 4,8-dioxo-6β-methoxy-7α,11-epoxycarabrane, and zedoarofuran [15]	Gastric cancer	An adjunct to allopathic medication [76]
*Curcuma zedoaria*	Temu putih	Rhizome	Curcumenone, Curcumenol [16]	Breast cancer	An adjunct to allopathic medication [77]

## 3. Sources of Curcumenol

Curcumenol was extracted from *Curcuma zedoaria*’s edible rhizome for the first time in the mid-20th century. In tropical forests, Zingerberaceae covers 52 genera, with 1500 species in total. Most of them are planted and reported in South East Asia [78]. Zingerberaceae, also known as “temu putih”, acts as a kind of additive; it is widely used in food preparation for nursing women after childbirth and as a spice in native dishes in Malaysia [79,80]. Curcumenol has also been identified in several other plants (e.g., the flower tea *Chrysanthemum indicum* [81], *Neolitsea pallens* [82], and *Torilis japonica* [83]). Zhu Shunying et al. [81] reported essential oils from samples of *Chrysanthemum indicum*, including monoterpene hydrocarbons (0.42–6.72%), sesquiterpenes (9.18–39.29%), and oxygenated monoterpenes (43.29–78.92%); curcumenol was derived from the oxygenated monoterpenes. G.C. Kharkwal et al. [83] investigated *Torilis japonica* (Houtt.) DC., which grows in the central Himalayan region. As revealed by their research, the oils are abundant in sesquiterpine hydrocarbons (approximately 60%). In total, 43 compounds with curcumenol accounted for 94.28% of the oils.

Rajendra C. Padalia et al. [82] investigated the composition of volatile constituents in the leaf, stem, and bark of *N. pallens* through nuclear magnetic resonance (NMR) spectroscopy, gas chromatography–mass spectrometry (GC–MS), and gas chromatography (GC) methods. Curcumenol (5.3%) was reported to occur in the oxygenated sesquiterpenoid, making up the major proportion of the bark oil. Curcumenol has also been extracted from bacterium and animal samples. Noor Akbar et al. [20] purified docosanedioic acid, Di-rhamnolipids, L-homotyrosine, N-acyl-homoserine lactone, and curcumenol from the gut bacteria of animals (e.g., cockroaches, water monitor lizards, and turtles).

## 4. Physicochemical Properties of Curcumenol

C_15_H_22_O_2_, 5β-Guaia-7 (11), 9-dien-8α-ol, 5, 8-epoxy-, 234.33, and 19431–84-6 are the molecular formula, chemical name, molecular weight, and chemical abstract service (CAS) number of curcumenol, respectively. Curcumenol has a white needle-like crystal structure at ambient temperatures, with a melting point of 113–115 °C, and it is insoluble in water, but soluble in methanol, ethanol, dimethyl sulfoxide, and some organic solvents. As indicated by the results achieved using spectroscopic and chemical methods, curcumenol exists in solution as a pair of hemiacetal–ketone tautomers [84]. More insights have been gained into the ultraviolet (UV) and electron-capture dissociation spectroscopic properties of curcumenol through theoretical time-dependent density functional theory calculations and modeling. Co-existing curcumenotone has a large tensional and unstable structure, contributing notably to the anti-oxidant process [84].

## 5. Extraction Methods

The extraction method for the components is of critical significance in the qualitative and quantitative process of herbal drug research [85]. Appropriate extraction methods should be developed to deal with different samples. Extraction methods for the different samples (e.g., biological samples, crude herb samples, and preparations) are detailed in Table 3.

A few investigations have reported on the extraction of curcumenol from biological samples. Salwa Mansur Ali et al. identified curcumenol and 11 other compounds from tissues and hemolymphs, which was extracted from the red-headed centipede, through the addition of protease inhibitors (serine/cysteine/metalloproteases) [86]. Subsequently, the samples were treated at 4 °C; then, ten cycles of freezing and thawing were performed on the gut and muscle tissues and, before analysis, the samples were subjected to sonication for further extraction [86].

**Table 3:** Sample preparation methods employed to extract curcumenol.

Matrix	Pretreatment/Extraction Approach	Procedure	Recovery (%)	Ref.
Biological Samples				
Crude hemolymph extract	Organ lysis	The exposed hemolymph was taken in an aseptic manner and suspended in a quantity of sterile distilled water. Ten cycles of freezing and thawing were applied to the gut and muscle tissues; then, they were homogenized, sonicated, centrifuged, and analyzed.	_	[86]
Plant				
Crude turmeric samples	Pressurized liquid water extraction	*Curcuma* Radix was dried at 60 °C and sieved with a 60-mesh sieve. The dried powder and diatomaceous earth were added to a stainless steel extraction cell. The extraction of the sample was obtained using the following parameters: 40% of the flush volume, methanol at 100 °C, static extraction for 5 min, 1000 psi.	_	[23]
Powdered rhizome	Ultrasonication in methanol	The powdered rhizome was extracted with methanol at 40 kHz, 200 W for 1 h using ultrasound, then filtered through 0.45 μm pores before analysis.	103.62	[87]
Powdered rhizome	Steam distillation	Powdered rhizome was exposed to vapor at 90 °C, followed by extraction for 30 min.	_	[88]
Powdered rhizome	Extraction with n-hexane	n-Hexane was shaken with samples for 30 min at 40 °C, and centrifuged at 4000 rpm.	_	[89]
Powdered rhizome	Pressurized liquid extraction	The extraction of the sample was achieved with the following conditions: static cycle, 1, filtering through a 0.45 μm icon filter for 40% of the flush volume; pressure, 1000 psi; static extraction period, 5 min; particle size, 0.2–0.3 mm; temperature, 100 °C, with methanol.	100.5	[90]
Different parts of the plant, powdered	Extraction with dichloromethane	Dichloromethane was added and the sample was macerated for 7 days at 15–20 °C.	_	[64]
Powdered rhizome	Cold immersion with methanol	A 0.5 g sample of powder was placed in a 100 mL triangle bottle, then 50 mL methanol was added, before cold soaking for 12 h, shaking every several minutes, until the supernatant was poured out.	99.6	[91]
Four sources of *Curcuma* Radix decoction pieces	Heating reflux with water	Decoction pieces were added in 10-fold amounts (m L/g) of water, soaked for 1 h, heated and reflowed twice, filtered, and concentrated.	116.8	[92]
Powdered rhizome	Ultrasonication in ethanol	Ultrasound with 70% ethanol for 45 min.	95.8–104.1	[93]
Powdered rhizome	Pressurized liquid extraction	Methanol; 120 °C; particle size, 0.2–0.3 mm; static extraction time, 5 min; pressure, 1500 psi; static cycle, 1; and 60% flush volume.	_	[94]
Formulation				
Decoction	Immersed in water and ethanol	Immersed in 50% ethanol, 70% ethanol, 100% ethanol, and water for 1 h each, then 2 h reflux.	_	[95]

In general, curcumenol is extracted from crude herb samples using various conditions and solvents (e.g., ultrasonication, pressurized liquid, and steam distillation) (Table 2). Zang Yuanfang et al. [96] developed an ultrasonic extraction method (power 600 W, frequency 40 kHz) with 70% methanol for 30 min, with a recovery rate of 99.8%. Yang F.Q. et al. indicated that a pressurized liquid extraction method could yield 90% recovery of curcumenol under methanol at 100 °C, with the particle size ranging from 0.2 to 0.3 mm, a static extraction time of 5 min, a pressure of 1000 psi, a static cycle, and a flush volume of 40% [97].

## 6. Methods for Qualitative and Quantitative Analysis

During the extraction of target compounds from different matrices, specific and effective analysis methods are also important. Curcumenol is a component of essential oils in many plant samples, and the accurate determination of volatile components in these samples is necessary. Thin-layer chromatography (TLC), colorimetry, HPLC, and GC with flame ionization detection or mass detection, have been used to perform quality control of essential oils in herbal medicines [98,99]. Nevertheless, the low separation efficiency of TLC and poor selectivity of colorimetry have hampered both their further application and development. A more powerful analysis tool was needed for the quality assessment of the abovementioned essential oils. Therefore, different qualitative and quantitative analysis methods have been established by researchers. Chromatography is a powerful and common tool used in research on curcumenol, involving different samples, covering GC and liquid chromatography (LC) (Table 4). For instance, Komatsu K. et al. [88] established a GC–MS method for the identification and detection of curcumenol in rhizomes of *C. kwangsiensis* from Guangdong Province, and *C. wenyujin* from Zhejiang Province, China, and *C. kwangsiensis* from the Guangxi Zhuangzu Autonomous Region (Guangxi A. R.). LC techniques have been widely applied for quantification in different fields, and are usually combined with other detectors (e.g., fluorescence, UV, and MS) [100,101,102]. F.Q. Yang et al. [90] developed a detection approach utilizing HPLC in combination with a diode array, with the aim of conducting rapid and simultaneous quantitative analysis of curcumenol and other compounds in the three species of *Curcuma* rhizomes. The measurement of the 11 above-described components in three different species of *Curcuma* rhizomes using the abovementioned method showed high repeatability, with inter-day and intra-day variances lower than 1.98% and 1.57%, respectively. It was effective in quantifying target components in samples of different *Curcuma* species. Gang Yin et al. [95] established UPLC-QQQ-MS to determine curcumenol and other bioactive components for the quality assessment of an AR–CR herb pair. The recovery (90.20–107.60%), repeatability (relative standard deviation (RSD) 5.98%), stability (RSD 4.29%), intra- and inter-day precision ((RSD) 3.64%, RSD 5.68%), limit of quantification (0.81–2.54 ng/mL), and limit of detection (0.33–10.78 ng/mL), were all used to optimize and validate the proposed method. Therefore, the primary bioactive ingredients in the AR–CR herb combination and the individual herbs were successfully compared using the established methodology.

**Table 4:** Spectroscopic and chromatographic methods used to assay the samples.

Analytical Method	Research Objectives/Title	Matrix and Sample Preparation Method	Result	Ref.
High-performance liquid chromatography (HPLC)	Determination of curcumenol in Yujin.	Powdered rhizome	Recovery value of curcumenol 103.62%.	[87]
HPLC	Simultaneous determination of 11 characteristic components in three species of *Curcuma* rhizomes using pressurized liquid extraction and HPLC.	Powdered rhizome	With intra- and inter-day fluctuations of less than 1.57% and 1.98%, respectively, this approach demonstrated good repeatability for the quantification of the abovementioned 11 components in three species of *Curcuma* rhizomes.	[90]
HPLC	Quantitation of Curcumenol extracted from water.	Four sources of *Curcuma* Radix decoction pieces	For *C. kwangsiensis*, *C. wenyujin*, *C. longa*, and *C. phaeocaulis*, the contents of curcumenol in the water extract were 0.066, 0.271, 0.058, and 0.310 mg/g, respectively.	[92]
Ultra-performance liquid chromatography (UPLC)	Determination of the curcumenol content in *Curcuma kwangsiensis*, vinegar-boiled *Curcuma kwangsiensis*, and water extract residues using UPLC.	Powdered rhizome and water residues	The established analysis method is accurate, stable, and sensitive; it can be used for the quality assay of *Curcuma kwangsiensis*, and it provides a scientific basis for the resource utilization of water extract residues.	[103]
High resolution gas chromatography (HRGC)	Quantitation of curcumenol.	Different parts of this plant, powdered	Standard samples of curcumenol within the concentration range of 0.03–0.93 mg/mL.	[64]
Liquid chromatography tandem–mass spectrometry (LC–MS)	Fingerprint of *Curcuma phaeocaulis* using LC–MS.	Powdered rhizome	Peaks 4–6 and 9 were identified as curcumin, demethoxycurcumin, curcumenol, and curcumone, respectively, through the contrast of the retention time and the online UV spectra and the molecular weight of the chemical standards.	[104]
Ultra-high-performance liquid chromatography coupled with the triple quadrupole tandem mass spectrometry method (UPLC-QQQ-MS)	Identification of curcumenol.	Decoction, dispensing granule decoction	It was the first time that 17 chemicals in the *Astragali* Radix–*Curcumae* Rhizoma (AR–CR) herb pair were simultaneously analyzed using UPLC-QQQ-MS, offering a workable approach in terms of overall quality control.	[95]
Ultra-high-performance liquid chromatography–quadrupole time-of-flight mass spectrometry	Identification of curcumenol.	Crude turmeric samples, pressurized liquid extractions	This new method led to the discovery of curcumenol and some other components as unique chemical markers for identification.	[23]
Gas chromatography–mass spectrometry (GC/GC–MS)	Identification of curcumenol.	Powdered rhizome	Identified curcumenol.	[88]
GC–MS	Identification of curcumenol.	Powdered rhizome	Two isomeric forms of curcumenol comprised the GC chromatogram’s major components, taking up 28.68 ± 0.91% and 17.96 ± 0.69% of the control plants’ total volatiles, respectively.	[89]
GC–MS	Identification and quantitation of curcumenol in *Curcuma* rhizomes.	Powdered rhizome	Curcumenol and four other compounds were optimized as markers for the quality control of *Ezhu*.	[94]
GC–MS	Determination of curcumenol in *Curcuma* rhizomes.	Powdered rhizome	Nine sesquiterpenoids were effectively quantified using the validated method in 18 samples of three *Curcuma* species, utilized as *Ezhu*.	[97]
GC–MS	Determination of curcumenol in the fractionation of volatile constituents originating from the *Curcuma* rhizome using GC.	Powdered rhizome	The structures of the compound were identified as curcumenol using a mass spectrometer (MS) and NMR spectra, respectively.	[105]

## 7. Other Factors Influencing the Content of Curcumenol

The contents of the components in plants are affected by their variety, place of origin, growth environment, processing methods, etc. [106,107,108,109]. Some factors relevant to the content of curcumenol (e.g., the constituents of minerals in the soil and the harvest season of herbs) have been illustrated. Rabia F. El-Hawaz et al. [89] examined the mineral concentration effects on the content of volatile constituents in rhizomes. The interaction of Ca^2+^ with KNO_3_ affected the contents of β-elemenone, isocurcumenol, germacrone, and curcumenol isomers I and II. The abovementioned findings demonstrated that minerals in the in vitro bioreactor medium during rhizome growth had an impact on the production of volatile components in turmeric when transferred to the greenhouse after six months. Christiane Regina Pamplona et al. [64] revealed that dihydrocurdione (2) and curcumenol (1) are two active terpenoids from different portions of *Curcuma zedoaria* cultivated in Brazil and that they displayed seasonal variations. As revealed by the results, the production of 1 and 2 was nearly three times greater in the mother rhizome in the autumn (Table 5 and Table 6). There are an insufficient number of studies focusing on the influencing factors of the content of curcumenol in *Curcuma wenyujin*. However, as a component of the volatile oil in *Curcuma wenyujin*, the content of volatile oil was affected by the areas of production [110], is shown in Table 7.

## 8. Bioactivity

Existing research has confirmed that curcumenol has numerous biological effects, including anti-tumor [13,14,15,16], anti-inflammatory [17,18], anti-virus [20,21], hepatoprotection [32], and neuroprotection activities [5]. Curcumin is another major bioactive substance contained in the *Curcuma* species; these two compounds have aroused increasing attention from chemists and biologists, and are expected to be investigated in-depth in the future. A comparison of the physiological effects of the two compounds is shown in Table 8.

**Table 8:** The physiological effects of curcumenol and curcumin.

	Curcumenol				Curcumin		
Disease/ Curcumenol Activity	Model	Treatment Doses	Mechanism	Ref.	Treatment Doses	Model	Ref.
Anti-inflammatory activity	BV-2 microglial cells	5–20 μM	Inhibiting Akt-dependent nuclear factor kappa-B (NF-κB) activation and the downregulation of Akt and p38 mitogen-activated protein kinase (MAPK) signaling	[18]	5–20 μM	BV-2 microglial cells	[111]
Anti-inflammatory activity	HaCaT cells	200 μg/mL	Inhibits the overexpression of inflammatory factors	[19]	20 μM	HaCaT cells	[112]
Analgesic effects	Mice	10 mg/kg	-	[64]	1000 mg/kg to 2000 mg/kg	Mice	[113]
Analgesic effects	Mice	12, 22, 29 μmol/kg	No involvement with the opioid system	[65]	-	-	-
Anti-oxidant ability and neuroprotective activity	NG108-15 cells	4 μM	-	[5]	25–100 μM	NG108-15 cells	[114]
Against coronary heart disease	Coronary heart disease rats	-	Improving blood lipid level, blood stasis, and myocardial infarction, and controlling the signaling pathway of PI3K/AKT/mTOR	[67]	-	-	-
Anti-diabetic activity	Human hepatocellular carcinoma (HepG2) cells	10 μM	-	[32]	-	Human	[115]
Ameliorating osteoporosis	Mice	100 μM	Impairs the stability of TRAF6 enhanced by IPMK and suppresses excessive osteoclast activity	[69]	100 mg/kg	Mice	[116]
SARS-CoV-2 infection	-	-	High-affinity interaction with proteins involved in coronavirus infection	[117]	10 µg/mL	DG614 strain and Delta variant	[118]
Anti-bacteriostatic effects	Gram-negative and Gram-positive bacteria	50 μg/mL	-	[20]	-	-	-
Anti-bacteriostatic effects	*P. capsici*	20 μg/mL	Damages the cell membrane	[21]	-	-	-
Breast cancer	MCF-7 cells	9.3 ± 0.3 μg/mL	Anti-proliferative activity and induces apoptotic cell death	[16]	20 μM	MCF-7 cells	[119]
Gastric cancer	AGS cells	263.34 ± 2.97 μM	Inhibition of proliferation	[15]	32 μM	AGS cells	[120]
Lung cancer	H1299 and H460 cells	100–400 μg/mL	Via the lncRNA H19/miR-19b-3p/FTH1 axis for lung cancer cell ferroptosis	[13]	5–30 μM	H460 cells	[121]
Cervical cancer	HeLa and C33A Cells	-	Through the tyrosine 3-monooxygenase/tryptophan 5-monooxygenase activation protein gamma polypeptide (YWHAG) pathway, facilitating cervical cancer cell apoptosis	[73]	20 μM	HeLa and C33A Cells	[122]
Inhibitory to cytochrome P450 (CYP) enzymes	Cytochrome P450 3A4 (CYP3A4)	12.6 ± 1.3 μM	-	[123]	11.93 ± 3.49 µM	CYP3A4	[124]
Binding to human serum albumin (HSA)	HSA	60 μM	-	[125]	-	HSA	[126]

### 8.1. Anti-Inflammatory Activity

Inflammation is the defense response of the immune system to tissue damage arising from trauma, infection, chemical exposure, etc. [127,128,129]. It is helpful in the repair of damaged tissues and removes harmful substances, but it is also destructive and disease may be triggered under excessive inflammatory conditions [130,131]. The production of inflammatory cytokines, followed by a decline in the biomechanics and structural integrity of the knee joint, results in osteoarthritis (OA) [132]. Xiao Yang et al. suggested that curcumenol reduced inflammation in ATDC5 chondrocytes and primary mouse chondrocytes, and ameliorated OA in a mouse model of medial meniscus-induced destabilization [17]. They employed tumor necrosis factor alpha (TNF-α) and Interleukin-1β (IL-1β) to treat ATDC5 chondrocyte and primary chondrocyte cells to induce inflammation. Curcumenol inhibited the progression of inflammation through downregulation of the expression levels of matrix metalloproteinase-3 (MMP3), inactivation of the MAPK and NF-κB signaling pathways, and the amelioration of catalytic changes and degradation of the extracellular matrix. Curcumenol can, therefore, be adopted to treat OA and inflammatory-mediated neurodegenerative diseases. Jia Ye Lo et al. [18] investigated the regulatory effect of curcumenol on the lipopolysaccharide-induced inflammation of BV-2 microglia, and reported that it could downregulate the Akt and p38 MAPK signaling pathways by inhibiting Akt-dependent NF-κB activation. Thus, the expression of proinflammatory mediators and regulatory genes is downregulated. Wang Jiafeng [19] suggested that 10 μg/mL curcumenol and other active components in *Curcuma* could inhibit the excessive secretion of four inflammatory cytokines (IL-1β, interleukin-6, interleukin-8, and interleukin 33) induced by TNF-α to different degrees in the treatment of psoriasis. Moreover, IκB-α and p-p65 expression in the NF-κB pathway could be suppressed. As revealed by the above results, the possible mechanism in the treatment of psoriasis by curcumenol and other active components in *Curcuma* is the inhibition of the TNF-α pathway and the inducement of the NF-κB pathway; subsequently, the inflammation and excessive proliferation of epidermal cells in the affected area can be alleviated.

### 8.2. Analgesic Effects

*Curcuma* rhizomes have long served as analgesics throughout history [6]. Some researchers have conducted phytochemical and analgesic activity analyses and attempted to determine the antinociceptive active compounds of *Curcuma zedoaria* R. Br. over the past few years [64,65]. Navarro et al. [65] reported that curcumenol exhibited manifold more potent and dose-associated analgesic activities in mice when evaluated in several models as compared with reference drugs. However, the mechanism of curcumenol does not cover the participation of the opioid system, as revealed by insufficient effects in the hot plate test.

### 8.3. Anti-Oxidant Ability and Neuroprotective Activity

Oxidative stress has been reported to arise from mitochondrial dysfunction, and it is also considered one of the critical contributors to neurodegeneration [133,134]. Compounds extracted from *Curcuma*, ginger, *Ginkgo biloba*, cinnamon, and related plants are characterized by their preventive and therapeutic roles in neurodegenerative disorders [135,136,137,138,139]. Omer Abdalla Ahmed Hamdi et al. [5] investigated the effect of components from *Curcuma zedoaria* (Christm.) Rosc. on hydrogen peroxide-induced oxidative stress in NG108-15 cells. The results showed that when NG108-15 cells were exposed to H_2_O_2_ (400 μM) for 24 h, the cell viability decreased to 67.6%. If pretreated with curcumenol, the protective effect is 100% at 4 μM and 97.7% when the curcumenol concentration is increased to 30 μM. Therefore, curcumenol has neuroprotective effects. Curcumenol also showed anti-oxidant activity in the oxygen radical anti-oxidant capacity (ORAC) assay [5].

### 8.4. Coronary Heart Disease (CHD)

CHD is the most common cause of death globally [140]. The *Curcuma aromatica Salisb. rhizome* (CASR) has often been employed to treat CHD arising from blood stasis syndrome in clinics, exhibiting multi-component and multi-pathway effects [67]. Chenghao Fei et al. [67] suggested that CASR is capable of significantly improving blood stasis, myocardial infarction, and blood lipid levels, and regulating the PI3K/AKT/mTOR signaling pathway, in CHD rats. Lipidomics has further shown that CASR can control the metabolism of glycerophospholipids, aberrant sphingolipids, and glycerolipids. Its effects may be attributed to (+)-curcumenol, curzerene, and isoprocurcumenol, which are reportedly the major active CASR compounds and are capable of mitigating vascular endothelial damage, upregulating blood lipid levels, and reducing blood viscosity [67]. These components could become promising drugs in CHD treatment.

### 8.5. Anti-Diabetic Activity

Over the past 20 years, obesity has spread over the world like wildfire, endangering lives by impacting nearly every organ system. It is currently a major public health concern and one of the most prevalent non-communicable diseases (NCDs) [141,142,143]. Changxin Zhou et al. [32] acquired extract compounds from *Curcuma wenyujin*, and adopted a glucose consumption model of HepG2 cells to evaluate their anti-diabetic effect. As the results showed, curcumenol and eight other compounds exhibited promising activity, with an over 45% increase in glucose consumption at 10 μM.

### 8.6. Ameliorating Osteoporosis

Osteoporosis is characterized by reduced bone mass and damaged bone microstructure; it is a systemic bone disease, which can result in bone brittleness and easy fracturing [144]. Despite substantial research into the pharmaceutical management of osteoporosis, better and more efficient treatment approaches are still needed [145]. Shiyu Wang et al. [69] revealed for the first time that curcumenol inhibits osteoclast differentiation in vitro and ameliorates osteoporosis in ovariectomy mice. Curcumenol can inhibit the protective effect of inositol polyphosphate multikinase on osteoclast differentiation, while hindering key downstream pathway activation when the receptor activator of nuclear factor-κB ligand induces osteoclastogenesis. Given the novel involvement of inositol polyphosphate multikinase in osteoclastogenesis and the possible therapeutic effect of curcumenol in regulating osteoporosis, inhibitors targeting inositol polyphosphate multikinase are likely to represent a novel direction for developing anti-osteoporosis medications.

### 8.7. Anti-Viral and Anti-Bacteriostatic Effects

With the rapid spread of the novel coronavirus disease over the past few years, further effective treatments are still required [146]. Gaurav S. Dave et al. selected curcumenol and three other potent candidates from natural compounds to treat and prevent coronavirus infection. Comparing the open state of severe acute respiratory syndrome coronavirus 2 to the closed state, curcumenol achieved an impressive docking score. This finding provides evidence in favor of the preventative ability of curcumenol to reduce the binding capacity of severe acute respiratory syndrome coronavirus in healthy humans [117].

As revealed by Noor Akbar et al. [20], the gut bacteria of animals that live in polluted environments serve as a promising source of anti-bacterial compounds. Using purified chemicals (e.g., curcumenol) extracted from the gut bacteria of such animals, these researchers determined the anti-bacterial and cytotoxic effects of these chemicals; cell viability tests were performed using lactate dehydrogenase release and methylthiazolyldiphenyl-tetrazolium bromide (MTT) assay, respectively. The findings showed that purified curcumenol and other compounds demonstrated significant anti-bacterial activities against several Gram-positive and Gram-negative bacteria, with effective minimal inhibitory concentrations of MIC50 and MIC90 at μg levels. Additionally, these concentration levels only exert minor effects on human cells, as indicated by the lactate dehydrogenase and MTT research [20].

As indicated by Wang Bi’s team, natural plant-derived zedoary turmeric oil (ZTO) (e.g., curcumenol) and several other components (curcumol, β-elemene, and curdione) serve as the main effective chemicals, exhibiting superb anti-fungal activity against *P. capsici* both in vitro and in vivo. It has been speculated that it exerts its effects by disrupting cell membrane integrity [21]. ZTO is a promising natural anti-fungal compound for the treatment of phytophthora blight arising from P. capsici.

### 8.8. Diversity of Anti-Cancer Effects

**Figure 2:**
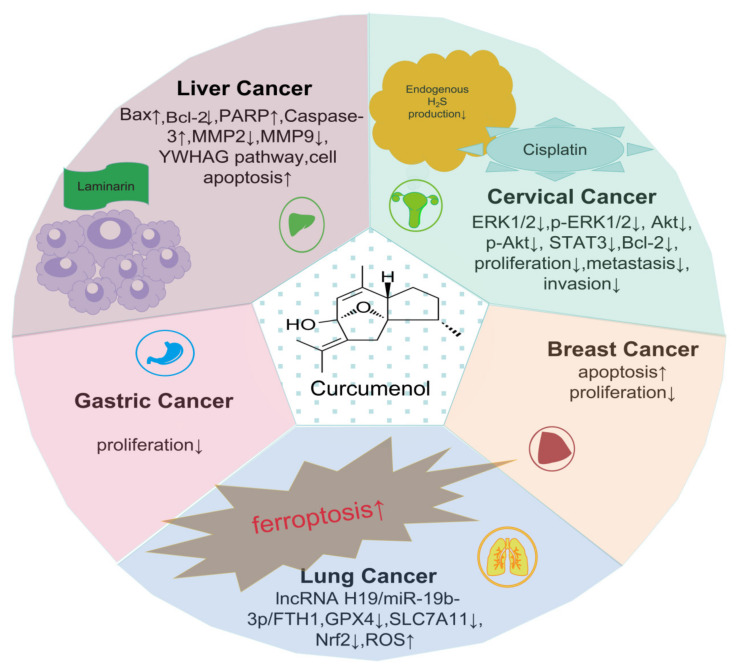
Diversity of anti-cancer effects. In this figure, we can conclude that curcumenol exerts five anti-cancer effects: suppressing breast tumor cells, digestive tumor cells, liver cancer cells, cervical cancer cells, and lung cancer cells. In cervical cancer, curcumenol can inhibit YWHAG expression in cervical cancer and reduce cervical cancer cell proliferation, invasion, and MMP2 and MMP9 expression when combined with cisplatin; the abovementioned combination of drugs leads to increased apoptosis. In liver cancer, the combination of curcumenol and laminarin can inhibit the proliferation, migration, and invasion of human hepatoma HepG2 cells. In lung cancer, curcumenol can suppress cell proliferation and induce cell death in lung cancer cells. Ferroptosis is the mechanism by which curcumenol induces lung cancer cell death; the lncRNA H19/miR-19b-3p/FTH1 axis was found to be crucial for this process. In gastric cancer, curcumenol has demonstrated the dose-dependent suppression of gastric cancer cell proliferation. In breast cancer, curcumenol showed remarkable anti-proliferative action against MCF-7 cells, which might cause apoptotic cell death. The increasing and decreasing expression of proteins were signed as ↑ and ↓, respectively.

Cancer is a large category of diseases that can occur in nearly every organ or tissue in the body, and is characterized by uncontrollable abnormal cell growth, invading adjacent parts of the body outside of the usual boundaries, and/or spreading to other organs. Chinese herbal medicines have long served as anti-cancer treatments, since they contain ample anti-cancer chemicals, exert promising cytotoxicity effects, regulate the tumor microenvironment and cancer immunity, and result in improved chemotherapy effects [147]. Existing research has suggested that curcumenol extracted from *Curcuma wenyujin* exhibits appreciable anti-tumor efficacy (e.g., suppressing breast tumor cells [148,149], digestive tumor cells [150], liver cancer cells [71], and lung cancer cells [13]), as shown in Figure 2.

#### 8.8.1. Effects on Breast Cancer

Breast cancer ranks highly among women with cancer-related mortality and is a major cause of death for women in their 40s [151,152]. Many researchers have identified effective compounds to treat breast cancer [153]. The chemical components in *Curcuma zedoaria*’s hexane and dichloromethane fractions were studied by Omer Abdalla Ahmed Hamdi et al. [16]. In total, 19 substances were examined using an MTT assay against Ca Ski, MCF-7, the human prostatic carcinoma cell line (PC-3), and HT-29 cancer cell lines, to determine whether they could inhibit cell proliferation. The two key ingredients of *Curcuma zedoaria*, curcumenone and curcumenol, both showed remarkable anti-proliferative action against MCF-7 cells, with the half-maximal inhibitory concentrations (IC50 values) reaching 8.3 ± 1.0 and 9.3 ± 0.3 μg/mL, respectively. As revealed by the results achieved based on the Hoechst 33342/PI double-staining experiment and phase contrast, they might cause apoptotic cell death. These findings underpin the ethnomedical use of *Curcuma zedoaria* as a breast cancer treatment.

#### 8.8.2. Effects on Gastric Cancer

Gastric adenocarcinoma ranks third in cancer-related deaths, with approximately 800,000 fatalities worldwide, and is the fifth most frequent malignancy in terms of incidence [154]. Although many chemical therapies exist, a new and effective candidate compound is still needed. Curcumenol has demonstrated the dose-dependent suppression of gastric cancer cell proliferation [15]. Curcumenol, 11-epoxycarabrane, 4,8-dioxo-6-methoxy-7, and zedoarofuran, exert lethal effects against stomach cancer AGS cells, with IC50 values ranging from 212 to 392 μM, as found by Tae Kyoung Lee’s research team. As the primary cytotoxic components in *C. zedoaria* rhizomes, curcumenol and three other compounds offer additional experimental support for the traditional usage of *C. zedoaria* rhizomes in treating gastric cancer.

#### 8.8.3. Effects on Lung Cancer

Lung cancer tumors have the highest mortality rate in the world; however, even though there have been many improvements in the therapy of lung cancer [155], developing new effective drugs is still urgently required. Zhang Ruonan et al. [13] reported that curcumenol could suppress cell proliferation and induce cell death in lung cancer cells. Their research showed that ferroptosis was the mechanism by which curcumenol induced lung cancer cell death, and that the lncRNA H19/miR-19b-3p/FTH1 axis was crucial for this process. In light of this, curcumenol could be used to treat lung cancer patients.

#### 8.8.4. Synergistic Anti-Cancer Effects

**Figure 3:**
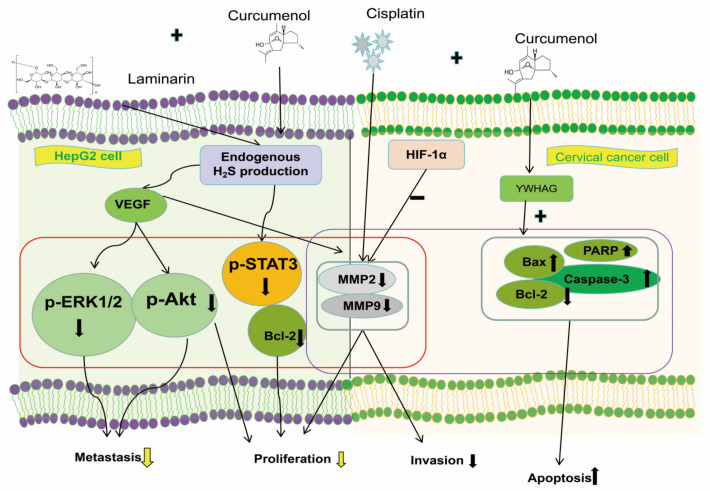
The mechanism of synergistic anti-cancer effects [71,73]. The combination of curcumenol and laminarin could inhibit the proliferation, migration, and invasion of HepG2 cells, and it may inhibit the angiogenesis of hepatoma via H2S and the VEGF pathway, consequently inhibiting the metastasis of HepG2 cells. In cervical cancer, curcumenol could inhibit YWHAG expression and reduce cervical cancer cell proliferation, invasion, and MMP2 and MMP9 expression when combined with cisplatin. The abovementioned combination of drugs led to increased apoptosis, downregulated Bcl-2 expression, and the upregulated expression of Bax, PARP, and caspase-3. The increasing and decreasing trend were signed as ↑ and ↓, respectively.

Drug combinations have been demonstrated to alleviate toxic side effects and improve efficacy in the treatment of cancer [156,157,158,159]. In addition to directly acting on different cancer cells, it has been reported that curcumenol can increase anti-tumor effects in combination with other anti-cancer compounds, such as cisplatin and laminarin [71,73]. Zhijie Mao et al. [73] determined that curcumenol extracted from *Curcuma* could inhibit YWHAG expression in cervical cancer and reduce cervical cancer cell proliferation, invasion, and MMP2 and MMP9 expression when combined with cisplatin, compared with cisplatin alone. The abovementioned combination of drugs led to increased apoptosis, downregulated B cell lymphoma 2 (Bcl-2) expression, and the upregulated expression of Bcl-2 antagonist X, polyadenosine diphosphate-ribose polymerase, and caspase-3. Accordingly, curcumenol is capable of increasing the anti-tumor effect of cisplatin against cancer cell proliferation, migration, invasion, and apoptosis. Curcumenol also exhibits enhanced anti-cancer effects against human hepatoma HepG2 cells when combined with laminarin. Huanxiao Han et al. [71] suggested that the combination of curcumenol and laminarin could inhibit the proliferation, migration, and invasion of human hepatoma HepG2 cells; the levels of pSTAT3 and Bcl-2 decreased in cystathionine beta synthase knockdown HepG2 cells. Furthermore, it can notably contribute to the use of kelp and *Curcuma zedoary* in liver cancer treatment. The mechanism of synergistic anti-cancer effects is shown in Figure 3.

### 8.9. Pharmacokinetics Study

Curcumenol is the key component of extensively employed natural products, requiring the determination of the pharmacokinetic parameters in order to guarantee safety and effectiveness. However, few studies have been conducted on its pharmacokinetic characteristics, as depicted in the following paragraphs (e.g., the interactions with human liver cytochrome P450 enzymes and the binding effect on human serum albumin).

#### 8.9.1. Inhibitory Effect on Human Liver Cytochrome P450 Enzymes

Curcumenol has been extensively used to inhibit cancer and inflammation, such that it is likely to be employed in combination with different drugs in many conditions to enhance treatment efficacy. It is imperative to evaluate the pharmacokinetics of drug–drug interactions induced by curcumenol. Dong-Xue Sun et al. [123] confirmed that CYP3A4 could be strongly inhibited (IC50 = 12.6 ± 1.3 μM) by curcumenol in the investigation of inhibitory effects on seven CYP isoforms. Curcumenol does not serve as a mechanism-based inhibitor, according to research into time- and nicotinamide adenine dinucleotide phosphate-dependent inhibition. However, given the limited pharmacokinetic information available, the promising clinical effects of curcumenol on patients are unlikely to have been adequately evaluated. Accordingly, more research should be conducted to determine the extent of the drug–drug interactions potentially arising due to curcumenol.

#### 8.9.2. Binding to Human Serum Albumin (HSA) In Vitro

The interaction of a compound with serum albumin can influence its pharmacokinetic characteristics, such as distribution, metabolism, elimination, and bioavailability, in bodily processes [160,161]. Based on this theory, Omer Abdalla Ahmed Hamdi et al. [125] investigated the binding of curcumenol and curcumenone to HSA through fluorescence quench titration. Molecular docking was performed to gain thorough insights into the interactions with HSA. Analysis of the fluorescence data indicated that there was a moderate binding affinity between the ligands and HSA. This showed that the binding constants of curcumenone and curcumenol were 2.46 × 105 M^−1^ and 1.97 × 104 M^−1^, respectively. The binding properties of curcumenol to HSA offer useful parameters for the detection of pharmacokinetic profiles based on the fluorescence spectroscopy and molecular docking findings. Nevertheless, since there are scant in vivo pharmacokinetic data, the pharmacokinetic parameters of curcumol, an analogue of curcumenol, in beagle dogs [150] can serve as a reference if required. However, specific pharmacokinetic data, in vitro and in vivo, remain to be obtained more substantially in the future.

### 8.10. Other Effects

In addition to the abovementioned bioactivities, further pharmacology effects have been identified. Curcumenol and some principal sesquiterpenes isolated from Zedoariae Rhizoma can exert prominent protective effects against liver injury mice [22]. Curcumenol may also be an effective candidate agent for treating structural and functional problems involving the skeletal muscle [84]. Zhang et al. [84] investigated the effect of curcumenol on myogenic differentiation and mitochondrial function. After increasing the mitochondria mass and function in myotubes, it was reported to promote the initiation of myogenesis and the formation of functional myotubes. Guanghui Zhong found that curcumenol could improve renal function in 5/6 nephrectomy rats with chronic renal failure [162].

**Figure 4:**
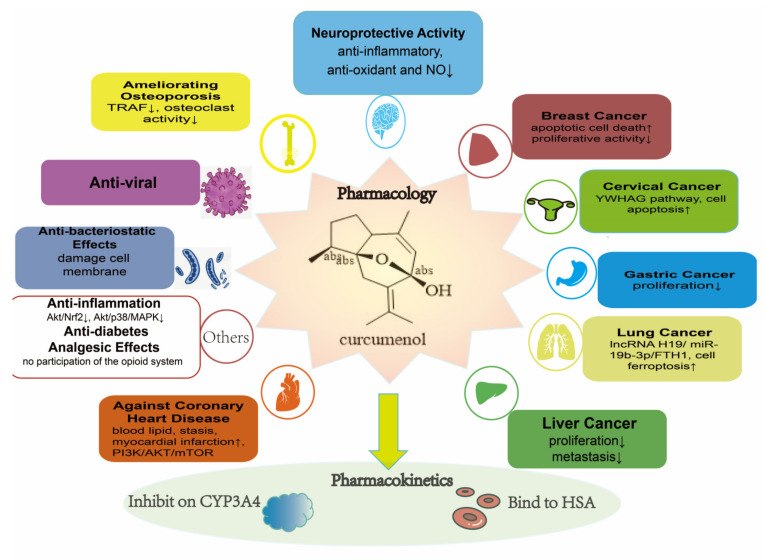
Biological activities of curcumenol. In this figure, we can conclude that the biological activities of curcumenol are very abundant, ranging from pharmacology to pharmacokinetics; in addition to anti-cancer effects, curcumenol exerts anti-inflammatory activity, analgesic effects, anti-oxidant ability, neuroprotective activity against coronary heart disease, anti-diabetic activity, osteoporosis-ameliorating, anti-viral activity, and anti-bacteriostatic effects. The anti-cancer mechanism is detailed in Figure 2. Neuroprotective effects, involving anti-inflammatory and anti-oxidant effects, and NO levels, were decreased. The amelioration of osteoporosis is associated with decreased TRAF expression and decreased osteoclast activity. Curcumenol has also exhibited anti-viral effects in various studies. The anti-bacteriostatic effects mechanism of curcumenol involves damaging the cell membrane. The anti-inflammation effect of curcumenol involves decreases in Akt, Nrf2, p38, and MAPK expression. It has also shown anti-diabetes effects and analgesic effects, but its mechanism does not involve the participation of the opioid system. The mechanism of action against coronary heart disease involves the PI3K/AKT/mTOR pathway. The increasing and decreasing trend were signed as ↑ and ↓, respectively.

Overall, curcumenol has immense value in the health field, as shown in Figure 4, and further in-depth investigations are warranted.

Although there are scarce pharmacokinetics data concerning curcumenol, it has shown an inhibitory effect on CYP3A, and binding effects to HSA, which are two key factors in the pharmacokinetics process in vivo.

## 9. Conclusions and Prospects

*Wenyujin*, a member of the *Curcuma zedoaria* family, has been reported to comprise a total of 169 compounds, thus far. It has a long history and has been extensively employed in TCM due to its abundant components, including curcuminoids, monoterpenoids, sesquiterpenoids, diterpenoids, etc. To elucidate the exact compounds that exert the abovementioned traditional curative effects using current medical theories and to determine the targets of its biological effects, so as to change the dosage form, improve the effect of treatment, and reduce the waste of TCM resources, many researchers have conducted studies and found a series of compounds related to those treatment effects, including curcumenol. To more effectively extract curcumenol from different plant samples, researchers have developed several methods (e.g., pressurized liquid water extraction, steam distillation, ultrasonication in methanol or ethanol, extraction with n-hexane, extraction with dichloromethane, cold immersion with methanol, and heating reflux with water). Different detection methods have also been established (e.g., HPLC, UPLC, HRGC, LC-MS/MS, and GC–MS methods) to precisely measure and identify traces of curcumenol. Curcumenol also exhibits extensive bioactive effects (e.g., anti-cancer, anti-inflammatory, anti-bactericidal, and anti-diabetic activities, and the amelioration of osteoporosis).

In traditional applications of *Curcuma*, it is often used as an adjuvant of pre-existing therapies with other Chinese medicine to treat diseases. Moreover, curcumenol, as an important active ingredient, can be combined with the main chemical components of other Chinese medicine compounds in preparations of clinical applications to achieve better clinical effects. In anti-cancer treatments, it should be combined with chemotherapy drugs to reduce the toxicity of these medications to normal cells, while enhancing the sensitivity of tumor cells to chemotherapy drugs, reducing the drug resistance of tumor cells, and reducing side effects. However, it can dissolve in organic solvents but is insoluble in water, which has seriously limited its pharmacological effects in basic research and clinical treatment. To address this, new preparations should be developed to increase the water solubility and biological target effects of curcumenol. It is also possible to modify the structure of curcumenol to increase its solubility and biological effects.

More pharmacokinetic parameters of curcumenol should be determined to establish its safety and efficacy, such that further references can be provided for applications in clinical practice. Furthermore, it could facilitate the development of TCM and safeguard consumers’ health and safety.

## Figures and Tables

**Table 5:** Content of curcumenol in various seasons and in a wide range of parts of *C. zedoaria* (mg/100 g dried plant) [64]. Reproduced with permission from Christiane Regina Pamplona, et al., Zeitschrift fur Naturforschung. C, Journal of biosciences; published by Verl. d. Zeitschrift für Naturforschung, 2006.

Part	Autumn	Winter	Spring	Summer
Roots	15.70 ± 0.14	8.90 ± 0.15	8.70 ± 0.12	1.5 ± 0.10
Mother rhizome	33.10 ± 0.12	9.10 ± 0.08	5.90 ± 0.05	6.0 ± 0.01
Rugous rhizome	10.40 ± 0.03	3.10 ± 0.03	2.00 ± 0.04	2.9 ± 0.03

**Table 6:** Content of dihydrocurdione in various seasons and in a wide range of parts of *C. zedoaria* (mg/100 g dried plant) [64]. Reproduced with permission from Christiane Regina Pamplona, et al., Zeitschrift fur Naturforschung. C, Journal of biosciences; published by Verl. d. Zeitschrift für Naturforschung, 2006.

Part	Autumn	Winter	Spring	Summer
Roots	8.50 ± 0.27	3.10 ± 0.15	4.40 ± 0.06	1.60 ± 0.10
Mother rhizome	25.00 ± 0.25	9.40 ± 0.17	6.10 ± 0.15	7.40 ± 0.14
Rugous rhizome	6.70 ± 0.03	2.90 ± 0.08	1.50 ± 0.06	1.50 ± 0.03

**Table 7:** Comparison of the content of volatile oil in *wenyujin* from different producing areas [110].

Producing Areas	Content of Volatile Oils
Taoshan Town, Ruian City, Zhejiang Province, China	2.0%
Mayu Town, Ruian City, Zhejiang Province, China	1.8%
Siqian Town, Wenzhou City, Zhejiang Province, China	1.5%
Yongjia County, Wenzhou City, Zhejiang Province, China	3.1%
Leqing City, Zhejiang Province, China	2.0%

## Data Availability

Data are contained within the article.

## Abbreviations

AGS cells	human gastric adenocarcinoma cells
AR	*Astragali* Radix
AR–CR	*Astragali* Radix–*Curcuma* Rhizoma
ATDC5 chondrocytes	mouse embryonic tumor cells
CAS	chemical abstracts service
CaSki	human cervical cancer
CASR	*Curcuma aromatica* salisb. rhizome
CHD	coronary heart disease
CR	*Curcuma* Rhizoma
CYP	cytochrome P450
CYP3A4	cytochrome P450 3A4
GC	gas chromatography
GC–MS	gas chromatography–mass spectrometry
MS	mass spectrometry
H460 cells	human large cell lung cancer cells
HaCaT cells	human immortalized keratinocytes
HeLa	human cervical carcinoma cell line
HPLC	high-performance liquid chromatography
HRGC	high-resolution gas chromatography
HSA	human serum albumin
HT-29	human colorectal adenocarcinoma cells
IL-1β	Interleukin-1β
LC-MS	liquid chromatography tandem mass spectrometry
MAPK	mitogen-activated protein kinases
MCF-7 cells	human breast cancer cell line
MIC	minimal inhibitory concentration
MMP	matrix metalloproteinase
MTT	methylthiazolyldiphenyl-tetrazolium bromide
NF-κB	nuclear factor kappa-B
NMR	nuclear magnetic resonance
OA	osteoarthritis
pSTAT3	signal transducer and activator of transcription 3 protein phosphorylation
PC-3	human prostate cancer cell
RSD	relative standard deviation
TCM	traditional Chinese medicine
TLC	thin-layer chromatography
TNF-α	tumor necrosis factor alpha
UPLC	ultra-performance liquid chromatography;
UPLC-QQQ-MS	ultra-high-performance liquid chromatography coupled to triple quadrupole tandem mass spectrometry method
UV	ultraviolet
YWHAG	tyrosine 3-monooxygenase/tryptophan 5-monooxygenase activation protein gamma polypeptide
ZTO	zedoary turmeric oil
